# TNF Blocking Therapies and Immunomonitoring in Patients with Inflammatory Bowel Disease

**DOI:** 10.1155/2014/172821

**Published:** 2014-03-18

**Authors:** Romain Altwegg, Thierry Vincent

**Affiliations:** ^1^Département d'Hépatogastroentérologie, Hôpital Saint-Eloi, Centre Hospitalier Universitaire de Montpellier, 80 Avenue Augustin Fliche, 34295 Montpellier, France; ^2^Département d'Immunologie, Hôpital Saint-Eloi, Centre Hospitalier Universitaire de Montpellier, 80 Avenue Augustin Fliche, 34295 Montpellier Cedex 5, France

## Abstract

Since their appearance in the armamentarium for inflammatory bowel disease (IBD) more than a decade ago, antitumor necrosis factor (TNF) inhibitors have demonstrated beneficial activity in induction and maintenance of clinical remission, mucosal healing, improvement in quality of life, and reduction in surgeries and hospitalizations. However, more than one-third of patients present primary resistance, and another one-third become resistant over time. One of the main factors associated with loss of response is the immunogenicity of anti-TNF biologics leading to the production of antidrug antibodies (ADAbs) accelerating their clearance. In this review we present the current state of the literature on the place of TNF and its blockage in the treatment of patients with IBD and discuss the usefulness of serum trough levels and ADAb monitoring in the optimization of anti-TNF therapies.

## 1. Introduction

Antitumor necrosis factor (TNF) biologics appeared over a decade ago in the armamentarium for inflammatory bowel disease (IBD). Originally evaluated in Crohn's disease (CD) and thereafter in ulcerative colitis (UC), their efficacy was demonstrated in both diseases and has deeply modified the management of patients with IBD [1]. Although they are potentially able to change the natural course of IBD and to decrease the need for surgery, absence or loss of response is frequent and only one-third of patients remain in clinical remission at 1 year [2]. Clinical response, steroid-free remission, and mucosal healing have been correlated with drug trough levels [3, 4]. However, anti-TNF pharmacokinetic is characterized by a considerable interindividual variability and antidrug antibodies (ADAbs) have been identified as one of the major factors impacting their clearance [5]. Thus, serum trough levels and ADAb measurement have been proposed for the monitoring of anti-TNF drugs and algorithms were defined for the management of patients with IBD [6].

## 2. Role of TNF in IBD Pathophysiology

While the etiology of IBD is still unknown, it is thought to involve complex interactions between genetic disposition, environmental conditions, life style, and microbial and immune factors resulting in a deregulated and excessive immune response directed against components of the normal microflora. CD and UC have been associated with exaggerated T helper (Th) type 1 and Th2 responses, respectively. More recent studies demonstrated that tissue damages result from mucosal inflammation mainly mediated by proinflammatory Th1 and Th17 lymphocyte subpopulations and their respective proinflammatory effector cytokines. In the gut of CD patients, activated Th1 and Th17 cells produce IFN*γ* and IL17 (A and F), respectively, which stimulate macrophages and induce the production of other inflammatory cytokines such as IL-1*β* and TNF*α* that subsequently promote matrix metalloproteinases (MMPs) production by stroma cells and mucosal damage [7]. Thus, it is now widely accepted that TNF*α* plays a strategic role in IBD pathophysiology, at the cross talk of the different inflammatory pathways involved in gut mucosal inflammation [8]. Accordingly, most of the efficient biologic therapies developed so far in IBD aimed at neutralizing the proinflammatory activity of the TNF pathway. The effects of TNF*α* are known to be mediated by TNF receptor I (TNF-RI) or TNF-RII. Ligation of TNF-RI, which is expressed on a wide range of immune and nonimmune cells, results in NF-*κ*B activation, cytotoxicity, and induction of proinflammatory cytokines and chemokines as well as antiapoptotic peptides [9, 10]. The effects on T lymphocytes are mainly mediated by interaction of TNF*α* with TNF-RII inducing a costimulatory signal to TCR-mediated T cell activation, thereby increasing T cell proliferation, expression of T cell activation markers (CD25, human leukocyte antigen-DR, and TNF-RII), and secretion of inflammatory cytokines including IFN*γ* and TNF*α* [11]. Accordingly, anti-TNF are able to inhibit T cell activation resulting in a decrease of proliferation and cytokine secretion (IFN-*γ*, IL-13, IL-17A, and TNF*α*) of both CD4+ and CD8+ T cell populations derived from UC patients [12]. On the other hand, TNF*α* and TNF-RII are also able to activate and expand protective CD4(+)FoxP3(+) regulatory T cells (Tregs) and seem critical for the stabilization of their phenotype and function in the inflammatory environment of the lamina propria in a mouse model of colitis [13]. These contrasting effects of TNF*α* on effector versus regulatory T cells may explain unexpected and disappointing results obtained with anti-TNF in some autoimmune diseases such as multiple sclerosis [14]. Altogether, these data underline the complexity of TNF*α* function via TNF-RI or TNF-RII on the course of intestinal inflammation, due to different susceptibility of epithelial cells and effector or regulatory immune cells. As an illustration, in dextran sulfate sodium- (DSS-) induced acute colitis in BALB/c mice, TNF-RI ablation led to exacerbation of the disease with increased inflammation and intestinal damage, while TNF-RII deficiency had opposite effects [15]. Nonetheless, studies in patients with IBD have extensively demonstrated the efficiency of anti-TNF therapies which directly inhibit activation of effector T cells and sensitize them to Treg-mediated inhibition with final restoration of immune homeostasis, resolution of inflammation, and mucosal healing. Further studies are now required to better understand the respective protective and deleterious effects mediated by TNF*α* on immune and nonimmune cells through TNF-RI and TNF-RII in order to develop more specific inhibitors with potentially an increased efficacy and/or safety.

## 3. Anti-TNF Therapies in Patients with IBD

TNF*α* is the major target molecule of biologic treatments in CD and UC. Numerous randomized clinical trials and meta-analyses have demonstrated the efficacy of monoclonal antibodies against TNF*α* for both induction and maintenance of remission in both CD and UC [16–18]. Infliximab (IFX), a chimeric monoclonal antibody composed of human constant and murine variable regions, and adalimumab, a fully human monoclonal IgG1 anti-TNF antibody, demonstrated their efficacy for the control of disease activity and the induction of clinical remission and mucosal healing in luminal CD and UC both in children and adult patients [1, 19–25]. Several randomized clinical trials showed a better efficacy in inducing steroid-free clinical remission for a combination therapy with immunomodulators than anti-TNF monotherapy in CD and UC [26]. Moreover several studies established the use of infliximab and adalimumab in active fistulizing CD in adult patients [27, 28]. Certolizumab, a polyethylene-glycolated Fab' fragment of anti-TNF Ab, also produced significant clinical benefit and mucosal healing in adult patients with CD [29]. Recently, golimumab, a fully human monoclonal antibody to TNF*α*, was shown to induce and maintain clinical response in patients with active moderate-to-severe UC [30, 31].

However, although 60 to 80 percent of patients exhibit a good initial response to anti-TNF treatments (defined as a Crohn's Disease Activity Index (CDAI) decrease from baseline >70 points for CD and a decrease in the Mayo score of at least 3 points and at least 30 percent for UC), only one-third of patients are in clinical remission without steroids at one year (defined as a CDAI <150 for CD and a total Mayo score of 2 points or lower, with no individual subscore exceeding 1 point for UC) [18]. Consequently, 20 to 30 percent of patients require dose intensification or interval adjustment in order to maintain long-term clinical benefit and an average of 10 to 20 percent per year lose response [32–36].

## 4. Drug Monitoring of Anti-TNF Biologics

Despite the high effectiveness of anti-TNF in patients with IBD, more than one-third of patients present primary resistance, and another one-third become resistant over time [37]. Optimal clinical response required the maintenance of clinically effective drug concentrations, but the pharmacokinetic of anti-TNF is highly variable among patients and could be influenced by numerous factors including gender, body weight, associated treatments (immunosuppressants are known to increase anti-TNF trough levels), route of administration, serum albumin concentration, and systemic inflammation with a markedly decreased half-life in patients with severe disease [38–40]. However, the main factor impacting anti-TNF pharmacokinetic and efficacy over time is immunogenicity whereby antidrug antibodies (ADAbs) accelerate anti-TNF monoclonal Abs clearance and shorten their half-life [41, 42]. Although humanized (e.g., certolizumab) and fully human Abs (e.g., adalimumab and golimumab) are logically less immunogenic as compared with chimeric ones (e.g., IFX), they can all induce ADAbs targeting murine and/or variable domains of the monoclonal Ab. Other factors may promote immunogenicity such as genotype in a minority of patients and drug agitation or freeze-thaw cycles that can induce immunogenic protein aggregates (for review [43]). Contrastingly, prescription of maintenance therapy with concomitant immunomodulators and achievement of suitable trough drug levels have been shown to reduce the risk of ADAbs [44].

Several studies assessed IFX trough levels after induction treatment or during maintenance therapy as predictors of sustained clinical response and showed a significant correlation between low IFX trough levels and decreased clinical response in CD and UC adult patients [3, 4, 34, 45–47] and in children with UC [48]. In a recent prospective study of IBD patients who have developed secondary failure to IFX, Paul et al. have shown that the only factor associated with mucosal healing after IFX optimization was a significant increase in IFX trough levels [49]. Antibodies to IFX (ATIs) were described in up to 60% when IFX was used on an* ad hoc *basis in practice and in 10 to 20% of patients in randomized controlled trials of maintenance therapy [43]. ATIs were associated with loss of clinical response, deterioration of endoscopic activity, infusion reactions, and low serum IFX concentrations [5, 41, 44, 46, 50–52]. However, some studies did not observe significant correlation between trough levels of IFX and CD activity or between positivity of ATIs and loss of clinical response or deterioration of endoscopic activity [3, 4, 53–55]. These discrepancies could be explained by different methods of measurements for ATIs and IFX concentrations, by the short follow-up time in some studies, and by the lack of consensual optimal levels of IFX for prediction of efficacy.

There are fewer data for adalimumab, but some studies also described a positive association between serum adalimumab concentration and clinical remission in CD [56–58]. Furthermore, while fully human, antiadalimumab antibodies were described in 2.6 to 17 percent of patients treated for CD or rheumatoid arthritis and significantly associated with low serum adalimumab trough levels and decreased clinical response [56, 59–61]. The relationship between pharmacokinetic data and efficacy is less clear for adalimumab than IFX with considerable variability and overlap in serum concentrations between patients with and without remission [57]. However, in an observational study evaluating the efficacy of adalimumab in 168 active CD patients who failed to respond to IFX, long-term clinical benefit was significantly associated with higher serum trough concentrations and absence of ADAb [56]. A recent study using adalimumab maintenance therapy in 40 adult patients with CD or UC showed a significant association of high trough levels of adalimumab with clinical remission and mucosal healing. Antiadalimumab antibodies were associated with low trough levels of adalimumab and lack of mucosal healing [58].

There is so far no data concerning trough levels and antidrug antibodies for adalimumab in children and in all patients for certolizumab and golimumab.

Serum trough levels measurement to detect subtherapeutic drug concentrations and identification of ADAb (therapeutic drug monitoring or TDM) are the most relevant and useful parameters for the monitoring of anti-TNF drugs to facilitate informed decision making in IBD patients with secondary loss of response to TNF antagonists. The clinical utility of the immunomonitoring was evaluated in a retrospective study conducted on 155 patients with IBD and loss of response to IFX [6]. They showed that measuring IFX and ADAb concentrations may impact treatment decision in 73%. When ADAbs were detected, the switch to another anti-TNF molecule allowed a partial or complete response in 92% versus 17% for dose escalation whereas drug escalation was the most efficient strategy in patients with subtherapeutic IFX concentration (86% versus 33% of partial or complete response, resp.). They concluded that increasing anti-TNF doses is ineffective in patients with ADAb but appropriate in case of subtherapeutic drug concentration and proposed an algorithm for optimization of therapeutic strategy in IBD patients with loss of response to IFX based on ADAb and trough drug measurement [6].

Interestingly, in a prospective study examining the course of ADAb formation and the clinical relevance of its assessment in the followup of patients with rheumatoid arthritis, Bartelds et al. showed that, among patients positive for antiadalimumab Abs, 67% developed ADAbs during the first 28 weeks and almost one-third during the first month of treatment [62]. However and despite a poor clinical response, patients with ADAbs discontinued treatment only after 52 weeks of therapy indicating an important delay between ADAb appearance and treatment adjustment. Furthermore, early trough level measurement after induction might also have a prognostic value with IFX trough levels above 3.5 *μ*g/mL at 14 weeks being associated with a sustained therapeutic response [63].

On the other hand, supratherapeutic anti-TNF trough levels might also be associated with paradoxical inflammatory side effects such as psoriasiform eczema or arthralgia [64]. In such patients, lowering doses could be beneficial in terms of not only safety but also decrease of the cost for the healthcare payer.

Altogether, these data plead for the clinical and economical utility of early therapeutic drug monitoring in the management of patients receiving TNF inhibitors. In case of a loss of response with low trough level without ADAb, an intensified therapy with the same drug should be recommended by increasing doses and/or decreasing intervals and eventually adding an immunosuppressant. When low trough level is related to the presence of ADAbs, therapy should be switched within the anti-TNF class and if necessary to a drug with another mode of action [63]. The addition of an immunomodulator might also be able to induce a decrease in ADAb level and to restore clinical response [65]. Of note, clinical response can occur despite the presence of ADAbs as described recently in a retrospective study [66]. Continued maintenance therapy with IFX induced ADAb disappearance in two-thirds of these patients after a median of 4 infusions suggesting that continued anti-TNF treatment could be considered in patients with clinical response and first ADAb detection. Indeed, recent studies investigating the kinetics of ATI formation confirmed that ADAb secretion may be transient and disappeared over time in almost one-third of patients [67, 68]. Compared to nontransient ATI that appeared usually within the first 12months of therapy, transient ATI was detected throughout the duration of IFX therapy [68]. Patients with sustained ATI were more likely to discontinue IFX treatment compared with patients with transient ATI [67].

In a very recent study using a decision analytic model that simulated 2 cohorts of patients with CD who become resistant to anti-TNF inhibitors, Velayos et al. compared the effectiveness of empiric dose escalation versus testing-based strategy over a 1-year time period [69]. Although both strategies yielded similar rates of remission (66% versus 63%, resp.) and quality-adjusted life year (0.800 versus 0.801), the testing-based strategy was less expensive than empiric dose escalation ($31,870 versus $34,266, resp.). Similarly, Steenholdt et al. showed in a randomized controlled trial that a testing-based strategy using an algorithm designed to identify the mechanism leading to secondary loss of response to IFX is more cost effective than empiric dose escalation in patients with CD [70]. In the monitored arm, patients with low serum IFX and ATIs were switched to adalimumab, patients with low serum IFX without ATIs underwent dose intensification, and patients with high IFX trough levels with or without ATIs were switched to an out-of-class therapy or screened for an alternate cause of their symptoms. Compared to the current dose intensification strategy, individualized therapy substantially reduced average treatment costs per patient with similar clinical response rates.

Large prospective and randomized studies are still required to validate all these approaches in patients with IBD and clear dose toxicity/efficacy relationships have yet to be established for anti-TNF inhibitors.

Finally, we have to keep in mind that, in the absence of standardization, the numerous assays developed for serum trough levels and ADAb measurement (Table 1) exhibit variable performances that could explain discrepancies between studies and difficulties in establishing clear cutoff values. There are currently no defined gold standard assays for quantification of anti-TNF drugs and ADAbs. A recent study compared three in house or commercially available assays (ELISA, bridging ELISA, and RIA) developed for the analysis of IFX levels and ATIs [71]. There was a good correlation between IFX and ATI levels measured with all 3 tests. The sensitivity of the three assays to detect ATIs was comparable with a slight advantage for the RIA test which is less sensitive than ELISA to drug interference caused by the presence of IFX in the serum impeding the detection of low ATI concentrations. Nevertheless, discrepancies between the three assays were not rare and conclusions of the study were highly debated highlighting the high need for standardization [72, 73].

## 5. Conclusion

Since the advent of anti-TNF biologics more than a decade ago, they have demonstrated beneficial activity in induction and maintenance of clinical responses, mucosal healing, improvement in quality of life, reduction in surgeries and hospitalizations, and the treatment of extraintestinal manifestations of IBD. However, despite good overall initial effectiveness, a significant proportion of patients lose response over time mainly because of ADAb production and accelerated drug clearance. Although optimal treatment strategies remain controversial, therapeutic algorithms were proposed based on serum trough levels and ADAb monitoring in order to rationalize drug adjustment. For the future, a better understanding of the ambivalent protective and deleterious effects mediated by TNF*α* and its receptors on immune and nonimmune cells during IBD might be crucial for the development of more efficient and safe biological inhibitors.

## Figures and Tables

**Table 1:** Summary of the assays used in cited references for drug serum trough levels and ADAb measurement.

Assays	Company	References
ELISA	Prometheus Laboratories (San Diego, USA)	[3, 4, 6, 66, 70]
	Matriks Biotek Ltd. (Ankara, Turkey)	[45]
	Theradiag (Marne la Vallée, France)	[49, 58, 71]
	In house	[5, 50, 52, 56, 59, 61, 62, 65, 67, 68, 71]

Radioimmune assay (RIA)	Biomonitor A/S (Copenhagen, Denmark)	[46, 66, 70]
	In house	[51, 59, 60, 62, 71]

Homogeneous mobility shift assay (HMSA)	Prometheus Laboratories (San Diego, USA)	[67]
